# Calf cramp in children with Charcot-Marie-Tooth disease: searching for therapeutic targets

**DOI:** 10.1186/1757-1146-4-S1-O18

**Published:** 2011-05-20

**Authors:** Fiona Hawke, Monique Ryan, Robert Ouvrier, Joshua Burns

**Affiliations:** 1 Discipline of Paediatrics and Child Health, The University of Sydney, Westmead, NSW, 2145, Australia; 2 Podiatry Program, The University of Newcastle, Ourimbah, 2258, Australia; 3 Neurosciences Department, Royal Children’s Hospital, Parkville, 3052, Victoria; 4 Institute for Neuroscience and Muscle Research, The Children’s Hospital at Westmead, Sydney, NSW, 2145, Australia

## Background

Leg muscle cramps have been identified as the strongest independent predictor of worse quality of life in Australian children with Charcot-Marie-Tooth disease Type 1A (CMT1A). There is no accepted treatment for cramp in children with CMT and the cause of cramp is not well understood. Potential therapeutic targets should be carefully identified to direct clinical trials of interventions.

## Methods

81 children aged 2-16 years with CMT1A were recruited nationally through the Australasian Paediatric Charcot-Marie-Tooth Disease Registry. Body size and measures of strength, ankle range, foot posture, balance, agility, endurance, gait and neurophysiology were assessed at The Children's Hospital at Westmead (Sydney) and Royal Children's Hospital (Melbourne). Data analysis included Pearson product moment and Spearman rank correlation coefficients for normally and non-normally distributed continuous data respectively, and Fischer's exact test for dichotomous data.

## Results

Of the 81 children, 26 reported calf cramp and 1 child each reported toe, quadriceps or arm cramp. Calf cramp was associated (p < 0.05) with older age; stronger foot inversion, eversion, dorsiflexion and plantarflexion measured with a hand held dynamometer; and better performance in long jump. When adjusted for age, foot strength and long jump performance were not associated with cramp. Interestingly, stronger fist grip and worse performance on the 9-hole peg test (a measure of fine motor coordination) were associated with calf cramp and remained associated when accounting for age. Children who experienced calf cramp were also more likely to experience hand tremors (42% vs 16%; p=0.004). Calf cramp was not associated with body mass index, ankle dorsiflexion range, foot posture, balance, agility, gait parameters or nerve conduction velocity. Results of logistic regression modelling will be presented at the conference.

## Conclusions

No physical or neurophysiological lower limb measure was associated with calf cramp when accounting for age. The association between increased age and cramp may be due to disease severity and/or progression. This hypothesis is supported by the association of calf cramp with hand tremors and worse fine motor coordination, yet does not explain increased grip strength. Paradoxically, calf cramp is not associated with any lower limb measure of disease severity/progression.

